# Contemplative Practices and Later Life Memory Among Women

**DOI:** 10.1093/geroni/igaa057.508

**Published:** 2020-12-16

**Authors:** Nirmala Lekhak, Tirth Bhatta

**Affiliations:** 1 University of Nevada, Henderson, Nevada, United States; 2 University of Las Vegas Nevada, Las Vegas, Nevada, United States

## Abstract

Contemplative practices such as meditation, yoga, and prayer had been used as coping resources to reduce the adverse impacts of stressful life experiences. Despite emerging scholarship on the benefits of contemplative practices for cognitive health, scant research has examined the influence of such practices on both episodic and working memory among women in later life. While the use of private prayer outside of church or temple has been shown to have statistically significant positive effect on episodic memory among older adults, previous studies have relied on measures that fail to capture various aspects of meditative practices (e.g., mental imagery, relaxation). Drawing from the Study of Women’s Health Across the Nation (n=2245) conducted during 2006-2008, this study investigates the effect of contemplative practices (e.g. meditation / imagery / relaxation technique, yoga, and prayer) on episodic and working memory. Multivariate regression model estimates suggests women who either used meditation, imagery or relaxation techniques had significantly better episodic (b=0.61, p=0.001) and working memory (b=0.32, p<0.05) as compared to those who did not use those methods. Surprisingly, the influence of prayer on episodic memory was negative (b=-0.36, p<0.05), while the influence of yoga on memory was not statistically significant. Our findings underscore the need to incorporate meditative practices in non-pharmacological interventions that are designed to improve later life memory.

